# Patulin Detoxification by Evolutionarily Divergent Reductases of *Gluconobacter oxydans* ATCC 621

**DOI:** 10.1021/acs.jafc.4c12572

**Published:** 2025-03-11

**Authors:** Nadine Abraham, Edicon Chan, Xiu-Zhen Li, Honghui Zhu, Lili Mats, Ting Zhou, Stephen Y. K. Seah

**Affiliations:** † Department of Molecular and Cellular Biology, University of Guelph, Guelph, Ontario N1G 2W1, Canada; ‡ Guelph Research and Development Centre, Agriculture and Agri-Food Canada, Guelph, Ontario N1G 5C9, Canada

**Keywords:** patulin, mycotoxin, detoxification, Gluconobacter oxydans, short-chain dehydrogenase/reductase, aldo-keto reductase

## Abstract

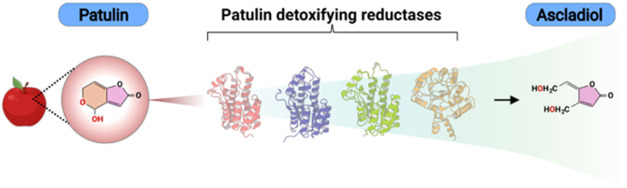

The mycotoxin patulin in processed apple juice poses a significant threat to food safety, driving the need for effective detoxification strategies. *Gluconobacter oxydans* ATCC 621 can detoxify patulin to ascladiol using either the short-chain dehydrogenases/reductases (SDRs)—GOX0525, GOX1899, and GOX0716—or the aldo-keto reductase (AKR) GOX1462. While GOX0525 and GOX1899 have been previously characterized, this study focuses on GOX0716 and GOX1462, evaluating their optimal pH, thermostability, thermoactivity, and substrate specificity, thereby completing the characterization of all four reductases. GOX0716 and GOX1462 exhibit pH optima of 6 and 7, respectively, and are functional across a broad temperature range of 25–55 °C. GOX0716 was determined to be more thermostable than GOX1462, with a half-life of 4.95 h at 55 °C. Phylogenetic analysis revealed that these SDRs belong to distinct evolutionary families with broad substrate specificity. GOX0716 is a member of the SDR79 family, which shares a common ancestry with the SDR111 family of fungal anthrol reductases. Conversely, GOX1462 is a member of the AKR18 family, which is involved in detoxification of the mycotoxin, deoxynivalenol (DON). Molecular docking analysis of Alphafold models highlights distinct variations in the active site architectures of these SDRs and AKRs, offering insights into their differing catalytic efficiencies toward patulin.

## Introduction

Patulin (4-hydroxy-4-H-furo[3,2-*c*] pyran-2[6H]-one) is a bicyclic polyketide mycotoxin produced by various species of filamentous fungi including *Aspergillus* sp.,^1^*Byssochlamys* sp.,^2^*Paecilomyces* sp.,^3^ and *Penicillium* sp.^4^ Among these, a prominent producer of patulin is *Penicillium expansum*, the primary causative agent of blue mold rot in apples.^5^ Patulin persistence in downstream apple products, such as juices, ciders, and purees, poses a serious food safety hazard. Acute exposure can elicit adverse gastrointestinal and immunosuppressive effects and neurotoxicity.^6^ This toxicity may be attributed to cellular oxidative stress^7^ by depletion of the antioxidant glutathione. It is proposed that this occurs via the Michael addition of sulfhydryl groups to the electrophilic α,β-unsaturated lactone ring of patulin.^8^ The toxicity of patulin may also be due to its ability to induce DNA damage^9,10^ and cross-linkages in proteins.^11^ Both the European Union (EU) and the United States Food and Drug Administration (FDA), therefore, enforce a limit of 50 ppb for fruit juices, 25 ppb for solid apple products, and 10 ppb for juices and foods destined for babies and young infants.^12^

To curtail patulin levels, the Food and Agriculture Organization (FAO) recommends the elimination of decayed apples.^13^ However, previous studies suggest that culling alone is insufficient as patulin may still be present in healthy portions of the fruit.^14^ Furthermore, apple juice processing stages can only partially reduce patulin levels in the finished product due to its thermostability^15^ and stability at low pH.^16^ Enzymatic detoxification of patulin is a promising means to reduce patulin levels in finished apple products and ensure consumer safety.

**Figure 1:**
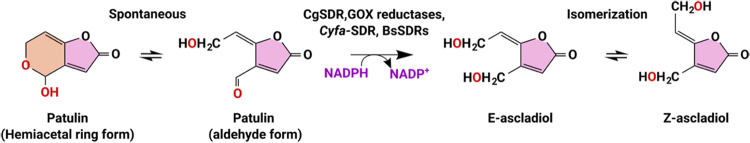
Patulin detoxification to ascladiol by recently isolated reductases.

Reductase enzymes that detoxify patulin have been isolated from yeasts, including *Candida guilliermondii*(17,18) and *Cyberlindnera fabianii*(19) and bacteria, such as *Gluconobacter oxydans*(20,21) and *Bacillus subtilis* 168.^22^ These microbes biotransform patulin to ascladiol, a metabolite that is non-cytotoxic in human cell lines.^23^ Mechanistically, this is proposed to involve a spontaneous opening of the hemiacetal ring upon which the resulting aldehyde form of patulin is enzymatically reduced to produce the E isomer of ascladiol (Figure 1). Spontaneous isomerization from E- to Z-ascladiol can also occur in the presence of sulfhydryl groups, as previously mentioned.^24^

The short-chain dehydrogenase/reductase (SDR) CgSDR, isolated from *C. guilliermondii*, achieved 90% transformation of 50 ppm of patulin to ascladiol within 72 h. However, 150 μg/mL enzyme was required, indicating a slow turnover rate.^17^ In contrast, *Cyfa*-SDR from *C. fabianii* achieved 98% degradation of 50 ppm of patulin within just 12 h using the same enzyme concentration.^19^ From *B. subtilis* 168, three SDRs, namely, BsSDR1, BsSDR2, and BsSDR3, were identified. Among these, BsSDR1 and BsSDR2 biotransformed 76.8% and 92.8%, respectively, of an initial patulin concentration of 23.1 ppm.^22^ Meanwhile, *G. oxydans* SDRs, namely, GOX0525 (10.3 μg/mL) and GOX1899 (47 μg/mL), demonstrated faster biotransformation rates at higher patulin concentrations (50 ppm) within 24 h under lower-pH conditions while requiring significantly lower enzyme amounts.^20^

*G. oxydans* is an attractive biocontrol agent and has a generally recognized as safe (GRAS) status, unlike certain strains of *C. guilliermondii* and *C. fabianii*, which are opportunistic fungal pathogens. *G. oxydans* are aerobic, Gram-negative, acetic acid bacteria that thrive in various ecological niches, ranging from fermented foods to fruits and flowers.^25^ They are important industry agents for vitamin C, gluconic acid, and vinegar production due to their ability to metabolize a range of substrates, namely, polyols, sugars, and sugar derivatives, through oxidative fermentation.^26,27^ Not surprisingly, they possess a large repertoire of evolutionary divergent SDRs and AKRs to attain this metabolic diversity, which makes them attractive candidates for industrial biotechnology, including mycotoxin detoxification.^28^

Two previously identified reductases were from *G. oxydans* ATCC 621, GOX0716 and GOX1462, have been shown to biotransform patulin into ascladiol. However, these enzymes have not been biochemically characterized due to difficulties in overproducing the enzymes in recombinant *Escherichia coli*. Here, we describe the conditions used to purify GOX0716 and GOX1462. We determined GOX0716 to be a member of the SDR79 family and GOX1462 to be an AKR18 family member. Both reductases are promiscuous and, in addition to patulin, display activity toward aliphatic aldehydes, aromatic aldehydes, and quinone compounds. A comparison of the active site of all four *G. oxydans* reductases with the substrate-bound crystal structure of yeast*C. guilliermondii* CgSDR (PDB ID: 7XWK) provided insights into how these evolutionarily divergent reductases utilize the same substrate, patulin, albeit with varying degrees of catalytic efficiencies.

## Materials and Methods

### Chemicals

Patulin was purchased from Cayman Chemical (Ann Arbor, MI). Ni^2+^-NTA Superflow resin was purchased from Qiagen (Mississauga, ON, Canada). Ascladiol was purchased from Triplebond (Guelph, ON, Canada). All other chemicals were obtained from Thermo Fisher Scientific (Toronto, ON, Canada) or Sigma-Aldrich (Oakville, ON, Canada) unless otherwise stated.

### Bacterial Strains

*G. oxydans* ATCC 621 was purchased from Cedarlane (Burlington, ON, Canada). Competent *E. coli* DH5α and *E. coli* LOBSTR BL21 (DE3) cells were purchased from Thermo Fisher Scientific and Kerafast, Inc. (Boston, MA), respectively.

### DNA Manipulation

The genes encoding GOX0716 and GOX1462 were PCR amplified from the genomic DNA of *G. oxydans* ATCC 621. The genes were inserted into the NdeI/*Hin*dIII sites of the pET-28a(+) plasmid and transformed into *E. coli* LOBSTR BL21 (DE3) cells as previously described.^20^

### Enzyme Purification

His-tagged GOX0716 and GOX1462 were recombinantly expressed in *E. coli* LOBSTR BL21 (DE3) (low background strain) (Kerafast, Inc.). An overnight starter culture was used to inoculate 4 L of LB media, and cultures were grown at 37 °C with shaking at 200 rpm. Recombinant protein expression was induced with 1 mM isopropyl β-d-1-thiogalactopyranoside (IPTG) and incubated at 15 °C overnight with shaking. Cells were harvested by centrifugation and washed with 20 mM HEPES pH 8.0.

The pellet was resuspended in 20 mM HEPES pH 8.0 buffer containing up to 1 mg/mL DNase I and lysed by 7–8 passages through a French press at 15,000 lb/in^2^. Cell debris was removed through centrifugation at 4 °C, and the clarified lysate was filtered through a 0.45 μm filter before incubation for 1 h at 4 °C with Ni^2+^-NTA resin in a buffer containing 50 mM sodium phosphate buffer (pH 8.0) and 300 mM NaCl, along with a binding buffer of 20 mM imidazole (pH 8.0) and 150 mM NaCl. The mixture was loaded onto a gravity column and washed with the same binding buffer. His-tagged proteins were eluted with 150 mM imidazole pH 8.0. Buffer exchange with 20 mM HEPES pH 7.5 with 10% glycerol and 150 mM NaCl was conducted in a stirred cell equipped with a YM-10 filter (Amicon).

Protein concentration was determined using a Bradford assay, with bovine serum albumin (BSA) as the standard.^29^ The purity of the recombinant enzyme and the molecular weights of recombinant GOX0716 and GOX1462 were estimated by using Coomassie blue-stained SDS-PAGE.

### LC–MS/MS Analysis

GOX0716 (1 mg/mL) was incubated with 300 μM reduced nicotinamide adenine dinucleotide phosphate (NADPH) and 2 mM patulin in 50 mM three-component buffer at pH 6.0 (0.1 M Tris, 0.05 M acetic acid, and 0.05 M 2-(N-morpholino)ethanesulfonic acid). GOX1462 was incubated with 300 μM NADPH and 2 mM patulin in 50 mM HEPES pH 7.0 buffer. Reactions were run overnight, and the reaction was quenched with 100% acetonitrile. The mixture was then filtered through a YM-10 filter.

LC–MS/MS analysis was conducted using a Thermo Scientific Q-Exactive Orbitrap mass spectrometer equipped with a Vanquish Flex Binary UPLC system (Waltham, MA). A Kinetex F5 100 Å column (150 × 4.6 mm^2^, 2.6 μm) (Phenomenex) was used for separation. The binary mobile phase consisted of solvent A (99.9% H_2_O/0.1% formic acid) and solvent B (99.9% acetonitrile/0.1% formic acid). The chromatographic elution conditions are as follows: 0–16 min, 5% B; 16–17 min, 5%–100% B; 17–21 min, 100% B; 21–22 min, 100–5% B; and 22–28 min, 5% B. The column compartment was kept at 22 °C; the flow rate was set at 0.3 mL/min; the injection volume was 10 μL; UV = 276 nm. The heated electrospray ionization (HESI) source was used in positive mode for the ionization of the target compounds; DDMS (top 10) mode was used with NCE set at 30.

### Phylogenetic Analyses

Amino acid sequences for homologues of GOX0525, GOX1899, GOX0716, GOX1462, and CgSDR were obtained through pBLAST. Maximum-likelihood phylogenetic trees were built in MEGA with 100 bootstrap replicates.^30^

### Molecular Modeling

The co-crystal structure of CgSDR (PDB ID: 7XWK) and the crystal structure of GOX0525 (PDB ID: 3WTB) were obtained from the PDB database. The Alphafold models of GOX1899 (Uniprot ID: Q5FPQ9) and GOX0716 (Uniprot ID: Q5FT03) were obtained from the Uniprot database. The 3D conformer of E-ascladiol was downloaded from the PubChem database (PubChem CID: 6440900) and edited using the Builder option in PyMOL (Schrödinger, Inc.) to generate the aldehyde form of patulin. The substrate was docked into the active site of each enzyme using the Rosetta Ligand docking protocol through the ROSIE server.^31−34^ Docked models were visually examined using PyMOL version 2.5.5, and the best representations were selected based on the following criteria: the C^3^=O of the aldehyde form of patulin was within hydrogen-bonding distance to the catalytic tyrosine and serine and within 3.0–4.0 Å distance from C^4^ of the nicotinamide ring.

### Tryptophan-Quenching Assays

Dissociation constants (*K*_d_) of GOX0716 and GOX1462 with NADPH were determined in triplicate using a tryptophan fluorescence-quenching assay with a PTI fluorimeter (with FelixGX software), as previously described.^35^

### pH Dependence

Enzyme assays were performed in triplicate at 25 °C using a Varian Cary 3 spectrophotometer with a thermojacketed cuvette holder. The pH dependence of enzymes was examined using a three-component buffer (0.1 M Tris, 0.05 M 2-(N-morpholino)ethanesulfonic acid (MES), 0.05 M acetic acid), covering a pH range from 4.0 to 8.0; the reaction mixture contained 250 μM NADPH and 2 mM patulin. Reaction rates of each enzyme were measured by monitoring NADPH oxidation at 340 nm with an extinction coefficient of 6220 M^–1^ cm^–1^. The rates of NADPH oxidation in the absence of the enzyme were subtracted from the measured rates. One unit (U) of enzyme is defined as the amount of enzyme required to oxidize 1 μmol NADPH per minute.

### Thermostability and Thermoactivity

Thermostability assays were carried out in triplicate by incubating aliquots of enzymes at 55 °C for 15, 30, 45, and 60 min. At the end of the incubation period, the enzyme aliquot was removed and cooled on ice for 1 min. Residual activity measurements were monitored at 25 °C with 250 μM NADPH and 2 mM patulin in the reaction mix. Data was fitted to a one-phase exponential decay equation to determine the half-life. For thermoactivity assays, aliquots of enzymes were preincubated for 5 min at a temperature range between 25 and 55 °C, which can be maintained using the water-circulating thermojacketed cuvette holder of the spectrophotometer. The prewarmed enzyme was then added to the reaction mixture containing 250 μM NADPH and 2 mM patulin. Reaction rates were monitored at each respective temperature.

### Substrate Specificity

For substrate specificity, kinetic measurements were performed at 25 °C in triplicate in 100 mM potassium phosphate buffer, pH 6.0 (for GOX0716) or pH 7.0 (for GOX1462). Substrate utilization was monitored by the decrease in the level of NADPH at 340 nm using a BioTek Epoch 2 microplate reader. Reactions were conducted in a total volume of 200 μL. The molar extinction coefficient of NADPH was determined by constructing a linear regression standard line using NADPH standards of known concentrations. One unit (1 U) of enzyme activity is defined as the amount of enzyme required to oxidize 1 μmol NADPH per minute. Stock solutions of substrates were prepared in dimethyl sulfoxide (DMSO). All kinetic data fitting was performed using nonlinear regression with GraphPad Prism, version 8. All data were fitted to the Michaelis–Menten equation.

## Results

### Purification of Recombinant GOX0716 and GOX1462

**Figure 2:**
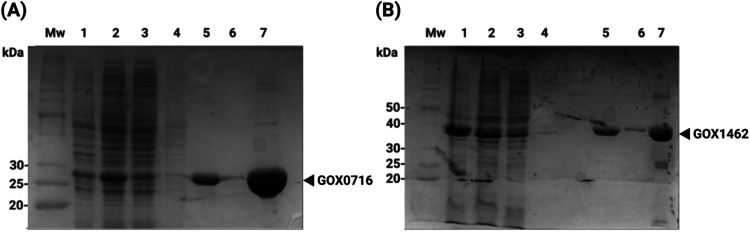
Coomassie blue-stained 10% SDS-PAGE gel showing fractions from Ni^2+^-NTA chromatography purification of recombinant (A) GOX0716 and (B) GOX1462 produced from recombinant *E. coli* LOBSTR BL21 (DE3). A benchmark protein ladder (Bio-Rad, Inc.) was used as a molecular weight marker (MW). Lane 1, insoluble fraction after lysis; lane 2, clarified lysate after lysis; lane 3, flow-through; lane 4, 20 mM imidazole elution fraction; lane 5, 150 mM imidazole elution fraction; lane 6, 250 mM imidazole elution fraction; and lane 7, concentrated protein from the 150 mM imidazole elution. The predicted MWs of GOX0716 and GOX1462 are 27.8 and 40.8 kDa, respectively, and the bands corresponding to each respective protein are indicated with an arrow beside the gel.

Previous attempts made to purify recombinant GOX1462 from *E. coli* resulted in protein aggregation, while GOX0716 was reported to be expressed in an insoluble form in *E. coli*.^20,36^ We reasoned that increasing the ionic strength and adding a chaotropic agent such as glycerol may improve the solubility of the enzyme. Therefore, following Ni^2+^-NTA chromatography of the recombinantly expressed N-terminal His-tagged protein, the enzymes were buffer-exchanged into 20 mM HEPES pH 7.0, 150 mM NaCl, and 10% glycerol. This reduced protein aggregation, and the yield of soluble GOX0716 was 19.7 mg of protein per liter of culture (Figure 2A), while that of GOX1462 was 2.74 mg of protein per liter of culture (Figure 2B). These values highlight the greater solubility of GOX0716 under optimized conditions.

### LC–MS/MS Analysis of Degradation Products Using Recombinant GOX0716 or GOX1462

**Figure 3:**
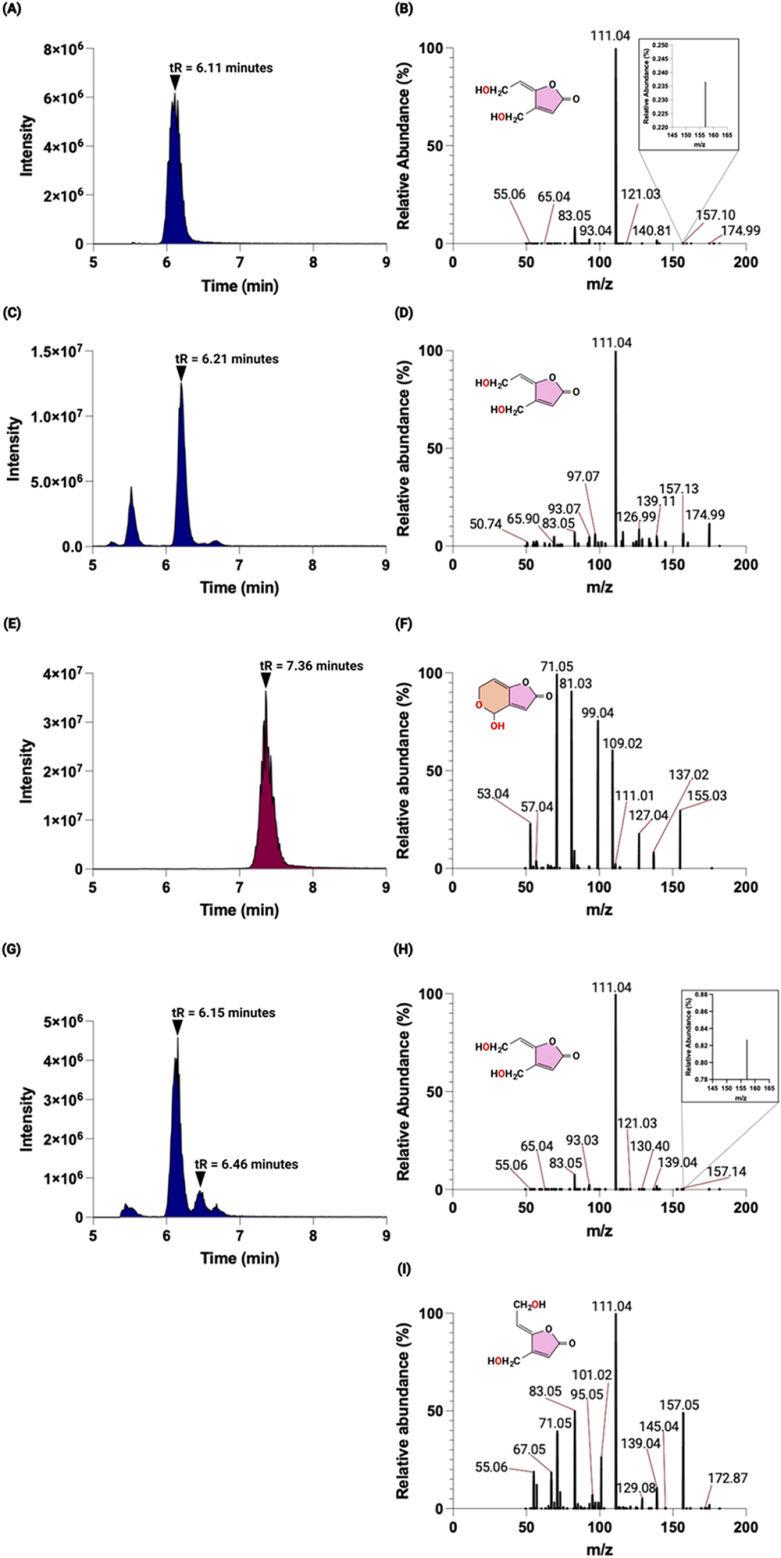
LC–MS/MS analysis of patulin biotransformation products. (A) For recombinant GOX0716, the total ion chromatogram reveals an analyte with a retention time, *t*_R_, of 6.11 min. (B) Mass spectrum of the analyte at 6.11 min showing an *m*/*z* of 157.10 corresponding to E-ascladiol. (C) Total ion chromatogram of the 10 ppm E-ascladiol standard with *t*_R_ = 6.21 min. (D) Mass spectrum of the 10 ppm E-ascladiol standard showing an *m*/*z* of 157.13. (E) Total ion chromatogram of the 20 ppm patulin standard with *t*_R_ = 7.36 min. (F) Mass spectrum of the 20 ppm patulin standard showing an *m*/*z* of 155.03. (G) For recombinant GOX1462, the total ion chromatogram reveals an analyte possessing *t*_R_ = 6.15 min and a second analyte with *t*_R_ = 6.46 min. (H) Mass spectrum of the analyte at 6.15 min showing an *m*/*z* of 157.14 corresponding to E-ascladiol. (I) Mass spectrum of the analyte at 6.46 min with an *m*/*z* of 157.05, suggesting that this is Z-ascladiol.

GOX1462 or GOX0716 (1 mg/mL) was incubated with 2 mM patulin in 300 μM NADPH overnight. The LC trace for the overnight reaction of GOX0716 with patulin showed the appearance of a peak with a retention time of 6.11 min (Figure 3A), and tandem mass spectrometry revealed a species with an *m*/*z* of 157.10 (Figure 3B). The characteristic of the analyte is consistent with the retention time and the mass spectrum of 10 ppm of E-ascladiol standard (Figure 3C,D). In contrast, a 20 ppm patulin standard had a retention time of 7.36 min (Figure 3E), and the mass spectrum revealed an *m*/*z* of 155.03 (Figure 3F), which is 2 amu less than E-ascladiol.

Likewise, for GOX1462, a peak with a retention time of 6.15 min was identified, which is confirmed to be E-ascladiol through tandem mass spectrometry (Figure 3G,H). A second minor peak with a retention time of 6.46 min was also identified. This peak shares the same UV spectrum as E-ascladiol and approximately the same *m*/*z* of 157.05 (Figure 3I). This peak is likely to be the Z isomer of ascladiol, which can be spontaneously produced from E-ascladiol and has been detected in prior studies involving *G. oxydans* M3.^21,24^

### pH Dependence of GOX0716 and GOX1462 Activity

**Figure 4:**
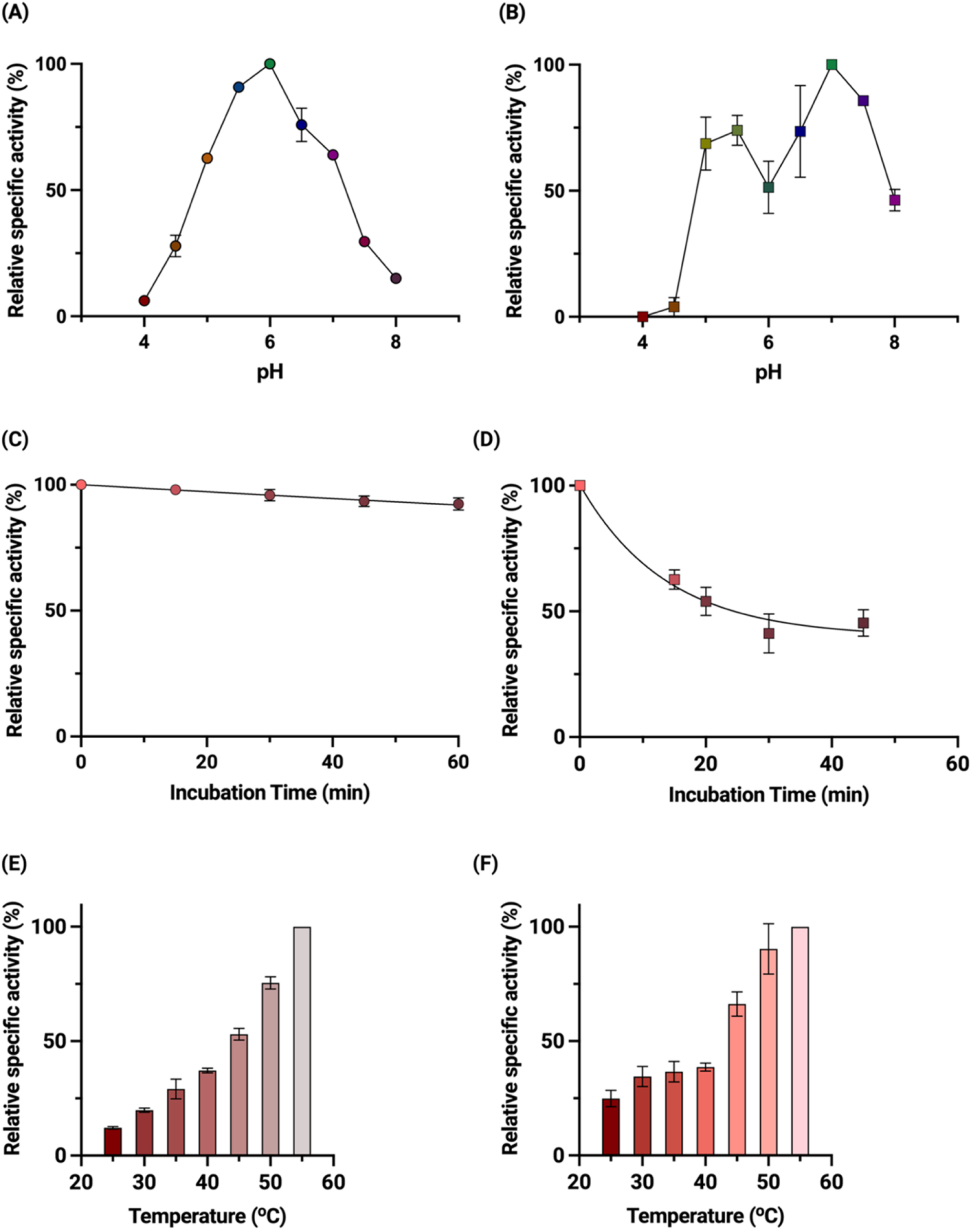
pH dependence, thermostability, and temperature activity range of GOX0716 and GOX1462. (A) pH dependence of patulin transformation activity of GOX0716. (B) pH dependence of patulin transformation activity of GOX1462. The assay consisted of 300 μM NADPH and 2 mM patulin in a tricomponent buffer containing 0.1 M Tris, 0.05 M MES, and 0.05 M acetic acid. (C) Thermostability of GOX0716. (D) Thermostability of GOX1462. Aliquots were removed at each time point, and the residual specific activity of the enzyme was determined spectrophotometrically at 25 °C. The assay consisted of 300 μM NADPH, 2 mM patulin in 0.1 M Tris, 0.05 M MES, and 0.05 M acetic acid pH 6.0 for GOX0716. For GOX1462, the same assay components were maintained, albeit the reaction was conducted at pH 7.0. (E) Effect of temperature on the activity of GOX0716. (F) Effect of temperature on the activity of GOX1462. Each reaction contained 300 μM NADPH and 2 mM patulin in either tricomponent buffer containing 0.1 M Tris, 0.05 M MES, and 0.05 M acetic acid at pH 6.0 for GOX0716 or 50 mM HEPES pH 7.0 for GOX1462. For all figures, the error bars indicate the standard deviation for each point performed in triplicate.

The activity of both enzymes was evaluated between pH 4.0 and 8.0. GOX0716 has optimal activity at pH 6.0, with a specific activity of 0.0107 ± 4.90 × 10^–5^ μmol min^–1^ mg^–1^ (Figure 4A). The enzyme activities were significantly lower below pH 4.5 and above pH 7.5. GOX1462 has optimal activity at pH 7.0, with a specific activity of 0.0346 ± 3.39 × 10^–4^ μmol min^–1^ mg^–1^, and it retains significant activity at pH 8.0 but, similar to GOX0716, shows low activity below pH 4.5 (Figure 4B). A reduction in specific activity was observed from pH 5.5 to 6. GOX1462 is prone to aggregation;^20^ therefore, a pH near its isoelectric point (predicted pI about 6) could reduce electrostatic repulsion between protein molecules that may exacerbate aggregation.

### Thermostability and Thermoactivity

Thermostability assays were conducted by incubating each respective enzyme for fixed time intervals (15, 30, 45, and 60 min) at 55 °C and measuring the residual enzyme activity at 25 °C. GOX0716 was highly stable at 55 °C, losing about 10% of its activity in 1 h (Figure 4C). The enzyme was also tested for thermostability at 80 °C but was unstable, with a half-life of less than a minute. GOX1462 was significantly less stable than GOX0716, with a half-life of 9.56 min at 55 °C (Figure 4D).

The temperature effect on enzyme activity was evaluated between 25 and 55 °C, a temperature range that can be maintained using the water-circulating thermojacketed cuvette holder of the spectrophotometer. Both enzymes retained high specific activity at 55 °C of 0.0420 ± 0.00068 μmol min^–1^ mg^–1^ for GOX0716 and 0.0311 ± 0.0023 μmol min^–1^ mg^–1^ for GOX1462. Both enzymes, therefore, can function at a broad temperature range (Figure 4E,F).

### NADPH Dissociation Constants

The dissociation constants (*K*_d_) for GOX0716 and GOX1462 were determined toward NADPH to quantify their binding affinity for this cofactor. For GOX0716, the apparent *K*_d_ for NADPH was 20.46 ± 2.86 μM, while for GOX1462, the apparent *K*_d_ for NADPH was 30.32 ± 3.56 μM.

### Substrate Specificity

The kinetic parameters for each enzyme were determined at their respective pH optimum. Both GOX0716 and GOX1462 were tested toward a range of aliphatic and aromatic aldehydes and quinone compounds (Table 1).

**Table 1:** Substrate Specificity Profiles of GOX0716 and GOX1462^a^

	**Enzyme**					
	**GOX0716**			**GOX1462**		
**Carbonyl substrate**	***K*_M,app_ (μM)**	***k*_cat,app_ (s^–1^)**	***k*_cat_/*K*_M,app_(M^–1^** **s^–1^)**	***K*_M,app_ (μM)**	***k*_cat,app_ (s^–1^)**	***k*_cat_/*K*_M,app_(M^–1^** **s^–1^)**
**Aliphatic aldehydes**						
Propionaldehyde	417 ± 52.1	0.0442 ± 0.00201	106 ± 14.1	164 ± 13.7	0.0228 ± 0.000523	139 ± 12
Butyraldehyde	351 ± 40.4	0.0339 ± 0.00135	96.6 ± 11.8	139 ± 14.4	0.0241 ± 0.000654	173 ± 18.5
Hexanal	509 ± 71.7	0.00946 ± 0.000516	18.6 ± 2.81	513 ± 141	0.0251 ± 0.00251	48.9 ± 14.3
Heptaldehyde	637 ± 72.8	0.0402 ± 0.00191	63.1 ± 7.81	178 ± 15.6	0.0270 ± 0.000671	152 ± 13.8
**α Keto aldehyde**						
Methylglyoxal	n.a.	n.a.	n.a.	1990 ± 238	0.221 ± 0.0159	111 ± 15.5
**Aldotriose**						
dl-glyceraldehyde	n.a.	n.a.	n.a.	483 ± 85.9	0.0181 ± 0.00121	37.5 ± 7.12
**Diose**						
Glycolaldehyde	607 ± 110	0.00643 ± 0.000459	10.6 ± 2.06	n.a	n.a.	n.a.
**Aromatic aldehyde**						
Benzaldehyde	n.a.	n.a.	n.a.	258 ± 37.7	0.0184 ± 0.000807	71.3 ± 10.9
**Di ketones**						
9,10-phenanthrenquinone	46.9 ± 8.17	3.14 ± 0.181	67,000 ± 12,300	31.9 ± 4.85	5.79 ± 0.234	182,000 ± 28,600
Isatin	282 ± 40.3	0.127 ± 0.00588	450 ± 67.6	107 ± 17.1	0.163 ± 0.00536	1520 ± 248
**Mycotoxin**						
Patulin	1300 ± 213	0.0468 ± 0.00402	36 ± 6.66	178 ± 25.4	0.0389 ± 0.00145	219 ± 32.3
**Furanic aldehyde**						
Furfural	94.8 ± 8.91	0.0103 ± 0.000223	109 ± 0.0964	n.a	n.a.	n.a.

a Apparent kinetic parameters for reduction of model aldehydes and ketones by recombinant GOX0716 and GOX1462 in the presence of 0.250 mM NADPH. n.a. indicates no activity detected.

Substrate specific assays revealed that both GOX0716 and GOX1462 were active not just with patulin but also toward a range of toxic endogenous and xenobiotic compounds. Overall, the best substrate for GOX0716 and GOX1462 was the xenobiotic compound, 9,10-phenanthrenequinone (9,10-PQ), although the catalytic efficiency (*k*_cat_/*K*_M_) of GOX1462 was 2.7 times higher than that of GOX0716. The substrate, 9,10-PQ, is a quinone compound derived from diesel exhaust particles that can trigger oxidative stress through redox cycling.^37^ Second, both enzymes displayed high catalytic efficiency toward the polyketone, isatin, with GOX1462 possessing a 3.4 times higher catalytic efficiency than GOX0716. GOX0716 was inactive toward methylglyoxal and dl-glyceraldehyde but exhibited low activity toward glycolaldehyde. Conversely, GOX1462 exhibited activity toward methylglyoxal and dl-glyceraldehyde. Additionally, it was also active toward aromatic aldehydes like benzaldehyde. Interestingly, we determined that GOX0716 was also active toward toxic furan compounds, such as furfural, which can occur in apple juices due to high pasteurization temperatures.^38^ Compared with patulin, the catalytic efficiency toward this substrate was 3.03-fold higher, making this compound a better substrate for GOX0716. A homologue of GOX0716, termed Cbei_3904 from *Clostridium beijerinckii*, was identified through transcriptome analysis and upregulated upon furfural-induced stress, supporting its biological relevance to furfural detoxification.^39^ In terms of patulin reduction activity, GOX1462 had a higher catalytic efficiency of 219 ± 32 M^–1^ s^–1^ than GOX0716 (36 ± 6.66 M^–1^ s^–1^). GOX1462 also possessed a 14.9-fold higher catalytic efficiency toward patulin than the recently characterized AKR DepB_Rleg_ from *Rhizobium leguminosarum*.^35^

Overall, both enzymes displayed broad activity toward aliphatic aldehydes. However, a slight preference for small-chain aldehydes like propionaldehyde and butyraldehyde over medium-chain aldehydes like hexanal and heptaldehyde was observed.

### Phylogenetic Analysis of Patulin-Detoxifying Reductases

Phylogenetic analysis was performed on all of the reductases that detoxify patulin in *G. oxydans* ATCC 621. GOX0525, GOX1899, and GOX0716 belong to the SDR superfamily, while GOX1462 belongs to the AKR superfamily. Both superfamilies consist of NAD(P)(H)-dependent oxidoreductases found in all domains of life with prominent roles in physiological and xenobiotic detoxification processes.

Although SDRs share low pairwise sequence identities (20–30%), they possess specific sequence motifs that define the coenzyme-binding site (GxxxGxG) and catalytic residues (NSYK).^40^ More than 300 SDR families have been identified and, based on their specific coenzyme-binding motifs, may be classified into seven subtypes. The largest among these are the classical (C) and extended (E) SDRs.^41^

**Figure 5:**
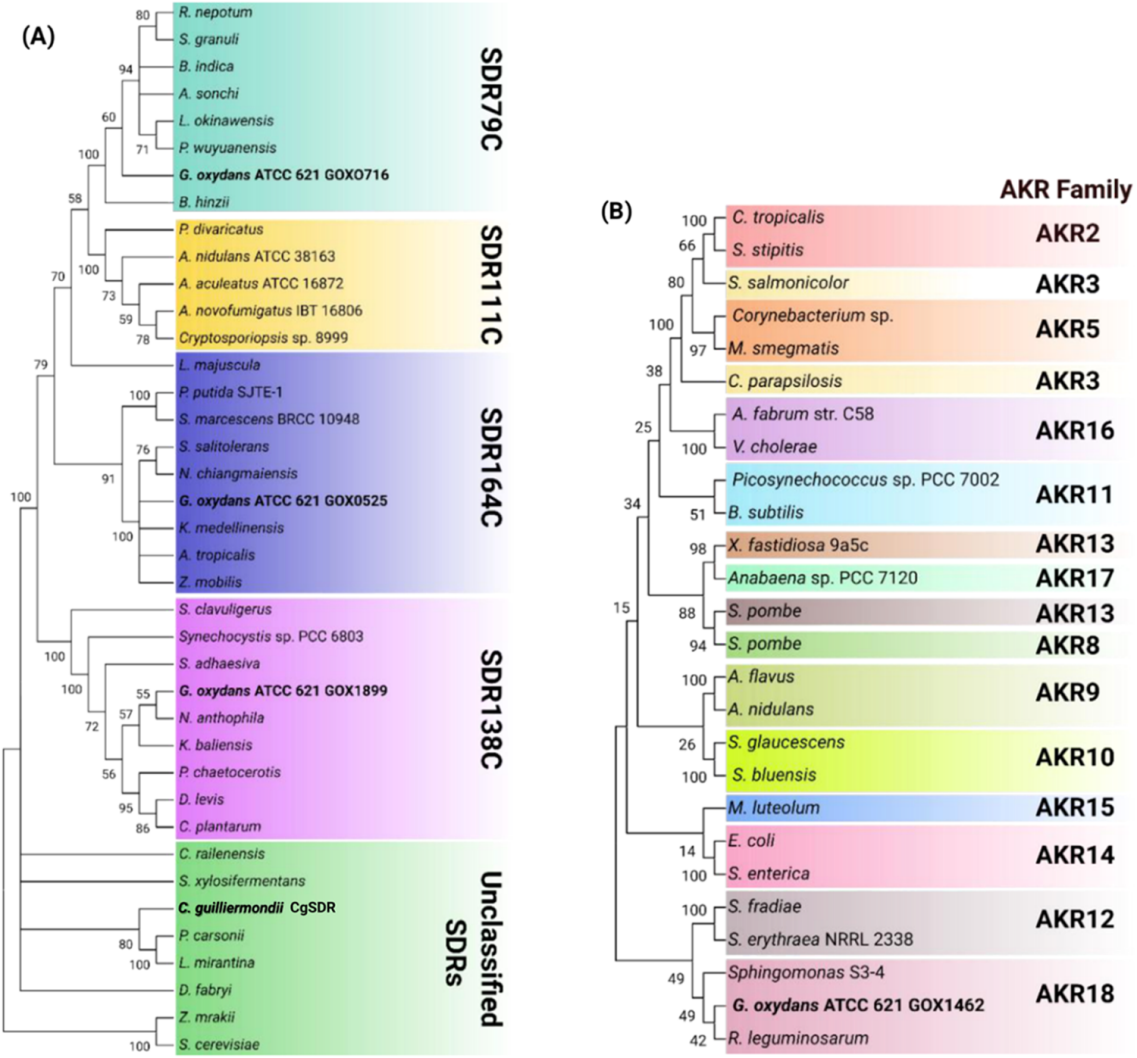
Dendogram of *G. oxydans* reductases involved in patulin detoxification. (A) Maximum-likelihood phylogenetic tree of homologues of *G. oxydans* SDRs. Each of these sequences was submitted to the short-chain dehydrogenases/reductases HMMR database for familial classification purposes (http://sdr-enzymes.org/). (B) Maximum-likelihood phylogenetic tree of homologues of GOX1462. For homologues of GOX1462, sequences were obtained for each family from the AKR Superfamily Database (https://akrsuperfamily.org/). The bootstrap consensus tree for GOX reductases inferred from 100 replicates was taken to represent the evolutionary history of the taxa analyzed. Branches corresponding to partitions reproduced in less than 50% of bootstrap replicates are collapsed. The percentages of replicate trees in which the associated taxa clustered together in the bootstrap test 100 replicates are shown next to the branches. GOX0525, GOX0716, GOX1899, CgSDR (*C. guilliermondii* SDR), and GOX1462 are shown in bold. Evolutionary analysis was conducted in MEGA11.

The three *G. oxydans* SDR together with the SDR from *C. guilliermondii* (CgSDR) were determined to be classical SDRs. Phylogenetic analysis, however, revealed that these SDRs belong to evolutionarily divergent subfamilies. CgSDR is closely related to most SDRs and is an unclassified SDR subfamily. Its closest functionally characterized homologue is found in *Saccharomyces cerevisiae* (Uniprot ID: YKL071W), which has NADH-dependent activity toward the lignocellulosic-derived aldehydes, such as formaldehyde, furfural, glycolaldehyde, and benzaldehyde^42^ (Figure 5A).

GOX0716 is a member of the SDR79C family. Members of this family include NADPH-dependent furfural-reducing enzymes. Moreover, SDR79C members appear to have shared common ancestry with fungal anthrol reductases (SDR111C), which are involved in the synthesis of tricyclic aromatic polyketides.^43,44^ GOX1899 was classified into the clavaldehyde dehydrogenase family of SDRs (SDR138C), of which *Streptomyces clavuligerus* SDR is the best-characterized member (Uniprot ID: Q9LCV7). This homologue of GOX1899 reduces the unstable intermediate, clavulanate-9-aldehyde, to form clavulanic acid, a medically relevant β-lactamase inhibitor.^45^ GOX1899 also possesses a broad substrate specificity and can reduce toxic medium- and long-chain aldehydes.^46^ A general role in toxic aldehyde scavenging was therefore ascribed to this SDR. GOX0525, classified into the SDR164C family, shares homology with a putative 3-oxoacyl-acyl-carrier-protein (OACP) reductase SDR from *Serratia marcescens* BCRC 10948. These proteins catalyze the reduction of OACP to (3R)-3-hydroxy-ACP during fatty acid biosynthesis.^47^ GOX0525 has also been found to reduce aliphatic α-ketoesters such as ethyl 2-oxo-4-phenylbutanoate (OPBE) and β-ketoesters such as ethyl 4-chloroacetoacetate (COBE) (Figure 5A).^36^

Classification schemes for AKR family members rely only on pairwise sequence identities. Sequences are clustered into families based on sequence identities exceeding 40%, with subfamilies sharing more than 60% sequence identity. Based on these criteria, GOX1462 was classified into the AKR18 family (Figure 5B). Current members of this family include an AKR from *Sphingomonas* S3-4 termed AKR18A1,^48^ DepB from *Devosia mutans* 17-2-E-8,^49^ and DepB_Rleg_ from *R. leguminosarum*.^35^ This AKR family possesses broad substrate specificity toward a variety of carbonyl substrates, including 3-keto DON, an intermediate in the deoxynivalenol (DON) epimerization pathway.^35,48,49^

### Substrate-Binding Site of Patulin-Detoxifying SDRs

To gain further insight into the basis for patulin reduction in these evolutionarily divergent SDRs, the structures of these enzymes were compared. The crystal structures of CgSDR (PDB ID: 7XWI) and GOX0525 (PDB ID: 3WTB) are available, and the Alphafold models of GOX0716 and GOX1899 are available in the UniProt database.

**Figure 6:**
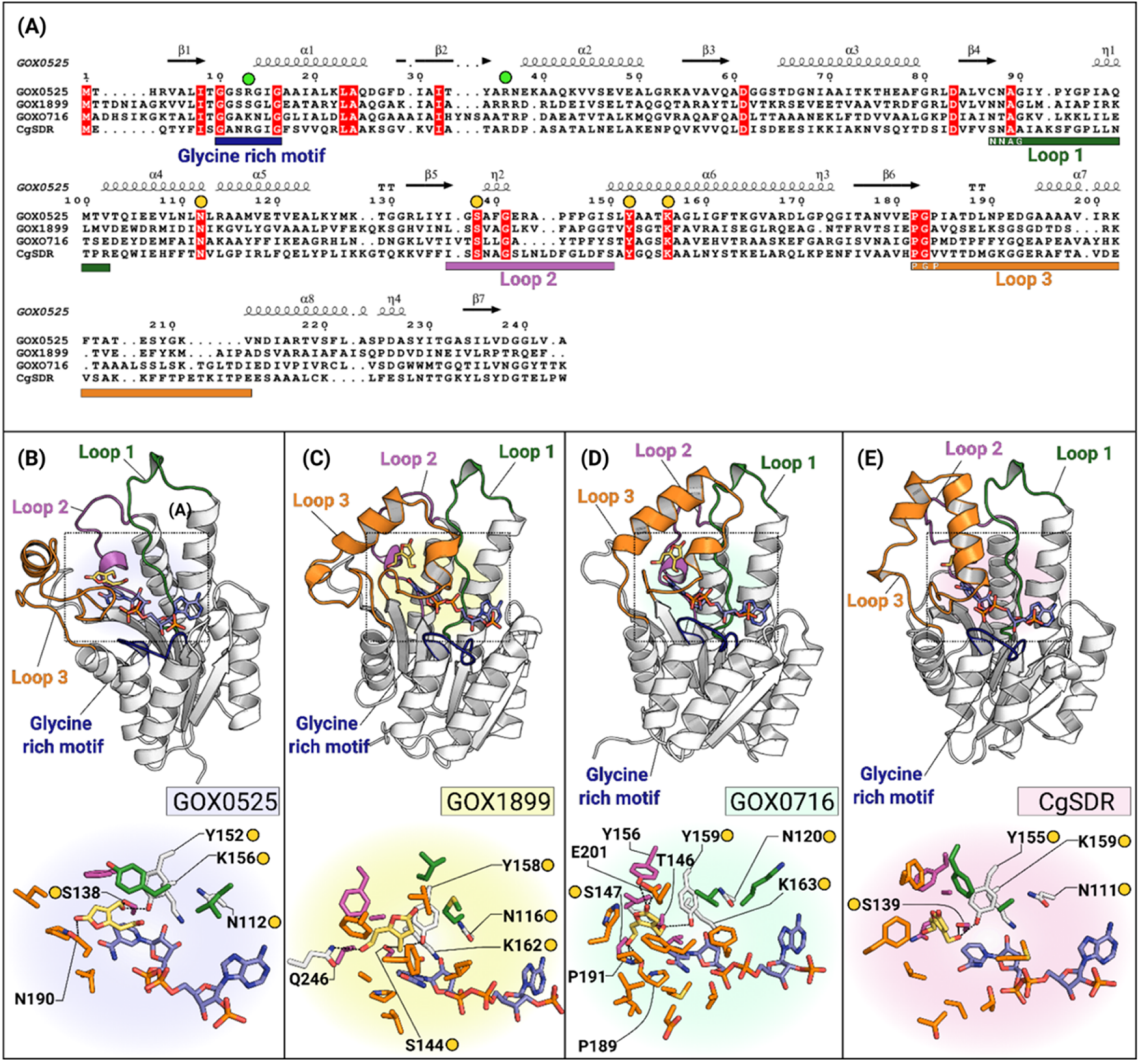
Multiple sequence alignment and docking results of GOX SDRs and CgSDR. Amino acid sequences included in the multiple sequence alignment (MSA) in panel (A) are as follows: GOX0525, GOX1899, and GOX0716 from *G. oxydans* ATCC 621 and CgSDR from *C. guilliermondii*. The conserved N-terminal glycine-rich motif has been highlighted indigo with residues involved in NADPH recognition indicated by green circles, while the conserved catalytic tetrad is indicated by yellow circles. Loop regions have been highlighted on the MSA along with relevant sequence motifs in white font and finally mapped onto the 3D structures of each respective SDR in panels (B–E). Active site residues involved in hydrogen-bonding and hydrophobic interactions with the aldehyde form of patulin (yellow sticks) are indicated for each reductase. Interacting residues in panels (B–E) are color-coded to match the loops they are located in.

All four SDRs share high homology in the N-terminal coenzyme-binding domain, known as the Rossmann fold, which consists of seven parallel β strands sandwiched between 3–4 α helices. NADPH-dependent SDRs normally have a basic residue preceding the second glycine in the nucleotide-binding glycine-rich motif (GxxxGxG), while a second basic residue is located at position 2 or 3 in the β2α2 loop. This is generally preserved in these NADPH-dependent patulin-detoxifying SDRs with either an arginine or a lysine in these positions. There are, however, some exceptions, with a serine in the glycine-rich motif of GOX1899 and a threonine in the β2α2 loop of GOX0716 (Figure 6A).

The variable C-terminal region of the SDRs comprises the substrate-binding site and houses the conserved catalytic tetrad consisting of tyrosine, serine, lysine, and asparagine. To gain insight into the interactions of the various SDRs with their ligands, the aldehyde form of patulin and NADPH were docked into the active site using Rosetta Dock.^31−34^ SDRs are stereospecific, involving 4-*pro*-S hydride transfer from NADPH with tyrosine acting as a catalytic acid, while a proximal lysine residue lowers the p*K*_a_ of tyrosine. The other catalytic residue, serine, stabilizes and polarizes the carbonyl functional group of the substrate, while asparagine completes the proton relay to bulk solvent.^50^ In all of the docked models, the C^3^=O of patulin aldehyde is within hydrogen bond distance to the phenolic and hydroxyl group of the catalytic tyrosyl and serine/threonine residues, respectively. Furthermore, it is also oriented for hydride transfer from NADPH.

The C-terminal active site of all four SDRs is encompassed by three loop structures, designated as loop 1 (β4η1α4), loop 2 (β5η2α6), and loop 3 (β6α7α8), that interact with the substrate (Figure 6A). In GOX0525, the C^3^=O of patulin aldehyde forms hydrogen bonds with the catalytic tyrosine, Tyr^152^, and Ser^138^ (loop 2) (2.7 and 2.8 Å). The O^4^ of the lactone ring also forms a hydrogen bond with the δ NH_2_ of Asn^190^ (loop 1) (Figure 6B).

In the GOX1899 model, the C^3^=O of the patulin aldehyde forms hydrogen bonds with the catalytic Tyr^158^ and Ser^144^ at distances of 2.9 and 2.8 Å (Loop 2). Additionally, a third hydrogen bond contact of 3 Å is present between the C^7^ OH of the patulin aldehyde and Gln^246^ (Figure 6C).

For GOX0716, the C^3^=O forms hydrogen bonds with the catalytic Tyr^159^ and Thr^146^ (loop 2) rather than serine at distances of 3.3 and 2.8 Å. Additional hydrogen bond contacts further stabilize interactions with the substrate. These include interactions between O^4^ of the lactone ring of the patulin aldehyde and Tyr^156^ (loop 2) and Glu^201^ (loop 3) and between the C^7^ OH group and the secondary amine and amide backbone of Pro^191^ and Pro^189^, respectively (loop 3) (Figure 6D).

In the CgSDR ternary complex, patulin binds in a hydrophobic cleft lined by residues Ala^90^, Phe^93^, Ser^139^, Leu^150^, and Phe^152^. Due to a lack of electron density in the loop 3 region of the crystal structure, residues 188–215 were not modeled. Hence, the Alphafold model of CgSDR was generated to reconstruct the missing loop region and was subsequently docked with the aldehyde form of patulin. Additional residues that were then identified include Phe^200^, Phe^211^, and Phe^212^, which may also contribute to hydrophobic interactions with the substrate (Figure 6E). Notably, a significant portion of the hydrophobic and polar interactions with our docked substrate are contributed by distinct amino acid residues in loop 3. Previously, site-directed mutagenesis was used to enhance the activity of CgSDR. The best mutant is the replacement of the loop 3 residue Val^187^ by Phe, confirming the importance of this loop for substrate recognition. The Val^187^ residue of CgSDR was replaced by Pro^184^ (GOX0525), Ala^189^ (GOX1899), and Pro^191^ (GOX0716) in the GOX enzymes. Another noticeable feature of the substrate-binding sites of these enzymes is their difference in both shape and size. Notably, GOX0525 has a large, solvent-exposed shallow active site relative to the other three SDRs, which possess slightly smaller, more enclosed active sites.

### Substrate-Binding Site of Patulin-Detoxifying AKRs

**Figure 7:**
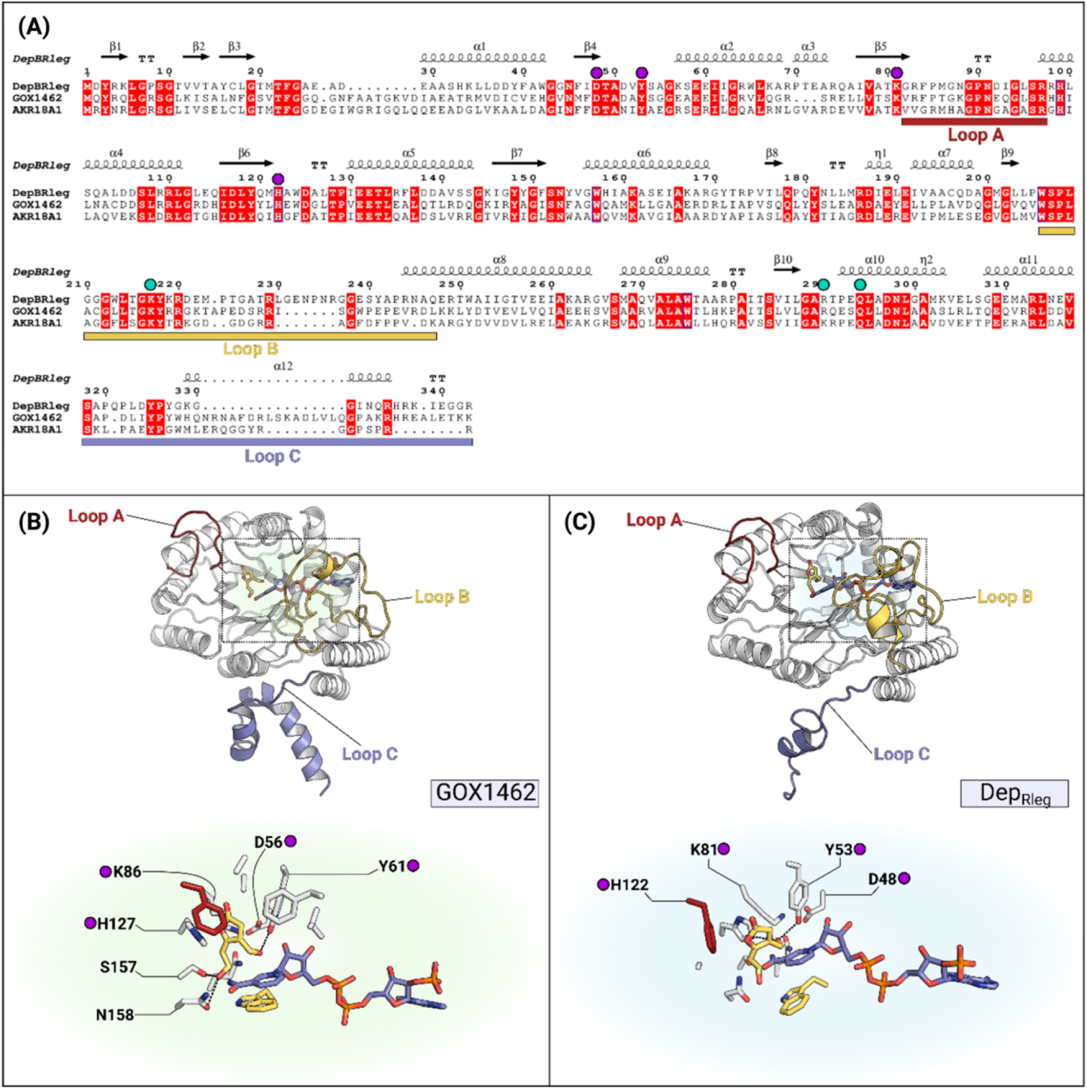
Multiple sequence alignment and docking results of GOX1462 and AKR18 family members. Amino acid sequences included in this alignment in panel (A) are as follows: GOX1462, DepB_Rleg_, and AKR18A1. Potential residues involved in NADPH recognition are indicated by cyan circles, while the conserved catalytic tetrad is indicated by purple circles. Loop regions have been highlighted on the MSA and mapped onto the 3D structures of each respective AKR. In panels (B) and (C), active site residues involved in hydrogen-bonding and hydrophobic interactions with the aldehyde form of patulin (yellow sticks) were presented for each reductase.

AKRs possess an eight-stranded parallel β-barrel, termed the triose phosphate isomerase (TIM) barrel (α/β)_8_ fold. Coenzyme binding occurs at the C-terminal end, and recognition of NADPH is facilitated via a combination of salt bridges and hydrogen-bonding interactions between 2′-monophosphate and proximal basic or hydrophilic residues (Figure 7A). Loop B, the coenzyme-binding loop, secures the NADPH in place, which leads to the reorganization of substrate-binding loops A and C.^51^ Much like SDRs, AKRs also follow an ordered bi-bi reaction mechanism; however, the stereospecificity of the reaction differs from the SDR catalytic mechanism. For AKRs, the reductive reaction involves a 4-*pro*-R hydride transfer from NADPH. Tyrosine, which comprises the catalytic tetrad (YDKH), acts as a general acid. The p*K*_a_ of this residue is lowered due to a proximal lysine and aspartate pair, which completes the proton relay to bulk solvent. The role of histidine in the catalytic tetrad is not as well understood, however, it has been proposed to be important for substrate orientation in the active site.^52^

The aldehyde form of patulin was docked into the Alphafold model of GOX1462 (Uniprot ID: Q5FQY5) and the Alphafold model of DepB_Rleg_ (Uniprot ID: J0WHR2) due to the absence of loop B in the crystal structure (PDB ID: 7UTF). The substrate-binding site of GOX1462 is lined with the following residues: Val^21^, Phe^29^, Asp^56^, Ala^58^, Ala^60^, Tyr^61^, Lys^86^, Arg^88^, Phe^89^, His^127^, Glu^128^, Ser^157^, Asn^158^, Gln^183^, and Trp^211^. In the docked model, C^3^=O forms one hydrogen bond with catalytic Tyr^61^, while C^7^ OH forms two hydrogen bonds with Ser^157^ and Asn^158^ (Figure 7B). In contrast, in DepB_Rleg_, the aldehyde form of patulin forms two hydrogen bonds only with catalytic Tyr^52^ and His^121^ (Figure 7C). Another noticeable feature about GOX1462 is the length of the β1α1 loop, which is eight residues longer than that of DepB_Rleg_. Phe^29^ from this loop extends toward the substrate-binding cleft and, together with Phe^89^, may play a role in securing the aldehyde form of patulin in place during catalysis.

## Discussion

*G. oxydans* possesses a vast repertoire of evolutionarily divergent reductases, making them valuable candidates for industrial biotransformation reactions. Four reductases from *G. oxydans* ATCC 621 can detoxify patulin to ascladiol. A summary table comparing the properties of all four GOX enzymes was compiled (Table 2).

**Table 2:** Comparison of Biochemical Properties of *G. oxydans* Reductases

**Enzyme**	**GOX0716**	**GOX1462**	**GOX0525**	**GOX1899**
**Catalytic efficiency toward patulin**(M^–1^ s^–1^)	36	219	1170	58.5
**Optimum pH**	6	7	6	5.5
T**hermostability(*****t***_**1/2**_**at55 °C)**	10% reduction in activity after 1 h	10 min	7 min	25% reduction in activity after 1 h

Among the enzymes tested, GOX0525 demonstrated the highest catalytic efficiency toward patulin, potentially addressing limitations in existing biotransformation systems that require large enzyme quantities, prolonged reaction times, or slightly acidic conditions to function. In terms of their optimal pH, GOX0525 and GOX0716 function best at a pH of 6.0, GOX1462 has the highest optimum pH activity at 7.0, and GOX1899 has the lowest optimum pH activity at pH 5.5.

Kinetic analysis showed that among all of the GOX enzymes, the catalytic efficiency of GOX0525 was 37.4 times higher than that of GOX0716 and 21.2 times higher than that of GOX1462. GOX0716 had the lowest catalytic efficiency toward patulin, followed by GOX1462. We determined that GOX0716 possessed activity toward furfural. Detoxification to furfuryl alcohol by substrain *G. oxydans* ATCC 621H has been reported elsewhere, but the enzyme responsible for this activity has not been identified previously.^53^ This is a promising feature of this enzyme since furfural, 5-methylfurfural, and 5-hydroxymethyl-2-furaldehyde are typically found as contaminants in apple juices and serve as markers for improper pasteurization.^54^ Therefore, this expands the substrate scope of GOX0716 besides patulin detoxification in apple juice. The thermostability of GOX0716 could potentially be utilized to detoxify furfural following the cooling of pasteurized apple juice. Furthermore, enzyme cocktails containing both GOX0716 and GOX0525 could be designed to tackle both patulin and furfural contaminations. However, the pH stability of these enzymes requires rational engineering approaches to shift the optimum pH. Surface redesign approaches could certainly be utilized to tailor proteins to exhibit higher net charges and minimize aggregation at desired pH conditions.^55^ Rational design of acid-tolerant protein variants has previously been demonstrated using the structure prediction software Rosetta Supercharge to alter the pH optima of glucose oxidase from pH 6.0 to 5.0.^56^ Similar strategies may be applied to modulate the pH optimum of the GOX enzymes in the future.

Phylogenetic analysis revealed that GOX0525, GOX1899, and GOX0716 all cluster in distinct SDR families, and they may not have specifically evolved for patulin detoxification, as evidenced by their varying degrees of catalytic efficiencies. Unlike GOX SDRs, the other patulin reductase, CgSDR, could not be classified into a specific SDR family, forms the furthest branch on the phylogenetic tree, and appears to be highly divergent. Among the enzymes characterized in this study, we determined that GOX0716 appears to be distantly related to SDR111C family members, which are fungal anthrol reductases.^44^ Their substrates are polycyclic aromatic hydrocarbons that resemble 9,10-PQ, which was determined to be the best substrate for GOX0716.

In terms of their 3D structure, SDRs possess an N-terminal Rossmann fold with a conserved glycine-rich motif (GxxxGxG) for binding dinucleotide coenzymes. Conversely, the C-terminal domain that binds the substrate shows sequence variability in the surrounding loops, which enables SDRs to utilize a broad range of substrates. Docking analysis suggests that the residues involved in interactions with the aldehyde form of patulin typically involve residues that lie downstream of specific SDR loop motifs, including the NNAG motif (loop 1), the catalytic serine (loop 2), and the PGP motif (loop 3) (Figure 6A). In addition, the majority of polar and hydrophobic contacts are made by residues located on loop 3. Due to the variability in sequences and lengths of these loops among the SDRs, the specific residues that interact with patulin in each enzyme are not conserved. Previous site-directed mutagenesis of CgSDR suggests that residues present in the PGP motif/loop 3 may be important for substrate enhancement studies, and potentially analogous residues in *G. oxydans* SDRs could be targeted in the future.

The other enzyme characterized in this study, GOX1462, is a member of the aldo-keto reductase (AKR) superfamily and, specifically, a member of the AKR18 family. Kinetic analysis showed that, like its homologue DepB_Rleg_, GOX1462 also reduces patulin to ascladiol using NADPH; however, its catalytic efficiency is at least 14.9 times higher. This improved catalytic efficiency is attributed to the lower apparent *K*_M_ and higher apparent *k*_cat_. Docking analysis showed that there are more stabilizing polar contacts between GOX1462 and the aldehyde form of patulin compared to those in DepB_Rleg_. Additionally, more hydrophobic contacts may be contributed by residues such as Phe^29^ that lie on the β1α1 loop.

In conclusion, this study showed that evolutionarily divergent reductases can detoxify mycotoxin patulin. GOX1462, the only identified AKR from this study with patulin reduction ability, was also noted to be a homologue of DepB_Rleg_, which is involved in DON detoxification. Enzyme cocktails consisting of mixtures of GOX enzymes, such as GOX0525 and GOX0716, could be designed to address both patulin and furfural contaminations in the industry. Although the pH of apple juice would limit the direct application of these enzymes, the enzymes can be utilized to detoxify patulin in water used to wash apples (flume water), where the neutral pH of this environment would sustain good catalytic performance. Wastewater biofilter systems utilizing whole *G. oxydans* ATCC 621 bacteria are other alternatives since cofactors like NADPH can be generated in situ and do not need to be exogenously supplied.

## Supporting Information Available

Table S1: Accession IDs for homologues of GOX SDR and AKR protein sequences utilized for the construction of dendograms (PDF)
