# Supplementary material for: Patulin Detoxification by Evolutionarily Divergent Reductases of Gluconobacter oxydans ATCC 621
Source: J Agric Food Chem. 2025 Mar 11;73(11):6842–53. doi: 10.1021/acs.jafc.4c12572 (PMC11926874; doi:10.1021/acs.jafc.4c12572)
Supplement: Supplementary file 1 — jf4c12572_si_001.pdf [file jf4c12572_si_001.pdf]

Supporting information

Patulin detoxification by evolutionarily divergent reductases of *Gluconobacter oxydans* ATCC 621

Nadine Abraham[1,2], Edicon Chan[1,2], Xiu-Zhen Li[2], Honghui Zhu[2], Lili Mats[2], Ting Zhou[2*], Stephen YK Seah[1*]

[1] Department of Molecular and Cellular Biology, University of Guelph, Guelph, ON, N1G 2W1, Canada

[2] Guelph Research and Development Centre, Agriculture and Agri-Food Canada, Guelph, ON, N1G 5C9, Canada

Corresponding Authors:

Stephen YK Seah

*Email: sseah@uoguelph.ca

Phone: 519-824-4120 Ext 56750

Ting Zhou

*Email: ting.zhou@agr.gc.ca

Phone: 226-971-3682

Table S1. Accession IDs for homologs of GOX SDR and AKR protein sequences utilized for construction of dendograms.

Enzyme	Organism	Accession number
**GOX0525**	*L. majuscule*	Q5V8A5
	P. putida SJTE-1	WP_014754112.1
	S. marcescens BRCC 10948	ANK79059.1
	S. salitolerans	WP_147093015.1
	N. chiangmaiensis	WP_077806211.1
	K. medellinensis	WP_014104635.1
	A. tropicalis	WP_061488448.1
	Z. mobilis	WP_012954776.1
**GOX1899**	*S. clavuligerus*	WP_003952516.1
	Synechocystis sp. PCC 6803	WP_010873912.1
	S. adhaesiva	WP_066711387.1
	N. anthophila	WP_211681834.1
	K. baliensis	WP_070405335.1
	P. chaetocerotis	WP_196474392.1
	D. levis	WP_161057097.1
	C. plantarum	WP_109584160.1
**GOX0716**	*R. nepotum*	WP_045021253.1
	S. granuli	WP_133036064.1
	B. indica	WP_012385737.1
	A. sonchi	WP_214007973.1
	L. okinawensis	WP_105861133.1
	P. wuyuanensis	WP_097120512.1
	B. hinzii	WP_029580610.1
**SDR111 (Fungal anthrol reductases)**	*P. divaricatus*	A0A411PQN6.1
	A. nidulans atcc 38163	Q5BH34.1
	A. aculeatus ATCC 16872	A0A1L9WLH9.1
	A. novofumigatus IBT 16806	A0A2I1C3T5.1
	Cryptosporiopsis sp. 8999	A0A4P8DJW8.1
**CgSDR**	*A. railenensis*	CAH2355868.1
	S. xylosifermentans	KAK6454289.1
	P. carsonii	XP_062810219.1
	L. mirantina	SCV01053.1
	D.fabryi	XP_015466496.1
	Z.mrakii	XP_037144018.1
	S. cerevisiae	YKL071W
**GOX1462**	*C. tropicalis*	BAA19477.1
	S. stipitis	CAA42072.1
	S. salmonicolor	AAB17362.1

	Corynebacterium sp.	AAA83534.1
	M. smegmatis	A0QV09.1
	C .parapsilosis	BAD01653.1
	A. fabrum str. C58	AAK90112.1
	V. cholerae	AAF96757.1
	Picosynechococcus sp. PCC7002	ABC69170.1
	B. subtilis	P46336
	X. fastidiosa 9A5C	AAF84538.1
	Anabaena sp. PCC7120	BAB74015.1
	S. pombe	Q09923
	S. pombe	O14295
	A. flavus	Q00049
	A. nidulans	Q00727
	S. glaucescens	CAA07384
	S. bluensis	AAD28516
	M. luteolum	BAC97800
	E. coli	AAA69168
	S. enterica	NP_461347
	S. fradiae	AAD41821
	S . erythraea NRRL 2338	AAB84068
	Sphingomonas S3-4	KY575150
	R. leguminosarum	J0WHR2
